# Effects of a cognitive stimulation program on physical and cognitive dimensions in community-dwelling faller older adults with cognitive impairment: study protocol

**DOI:** 10.1186/s12883-023-03154-1

**Published:** 2023-03-17

**Authors:** José Emanuel Alves, Lucas N. de Carvalho Pelegrini, Luana Rafaela Porcatti, Juliana Hotta Ansai, Maria Juana Beatriz Lima Candanedo, Karina Gramani-Say

**Affiliations:** Department of Gerontology, Federal University of São Carlos, Rodovia Washington Luiz, Km 235, São Carlos, São Paulo, 13565-905 Brazil

**Keywords:** Cognition, Elderly, Posture balance, Accidental falls, Clinical trial protocol

## Abstract

**Background:**

Cognitive functioning is an important dimension among the elderly. Cognitive maintenance is vital for aging due to its association with autonomy and independence. Considering the importance of preventive programs in older adults’ health, this study aims to share an intervention protocol of a falls prevention program for community-dwelling faller older adults with cognitive impairment.

**Methods:**

This is the protocol of an experimental and longitudinal study, consisting of cognitive stimulation associated with physical exercise in a 16-week fall prevention program. For cognitive intervention, the APG Cognitive Training Protocol will be used. Participants will be assessed pre-and post-intervention and will be randomly allocated to experimental or control groups. The screening protocol is composed of the TUG, FES-I, LAWTON & BRODY, ACE-R, GAI and fall survey instruments, focusing on the assessment of balance and mobility, fear of falling, performance on IADL, cognitive and anxiety tracking, respectively.

**Discussion:**

This study can determine the long-term effects of multimodal cognitive training, providing evidence for its replication in the provision of care for the elderly. The objective is to promote improvements in the cognitive performance, mobility and balance of the elderly, with a focus on reducing the number of falls, fractures, hospitalizations and institutionalization, serving as an alternative to interrupt the cycle of falls.

**Trial registration:**

The research was approved by the Research Ethics Committee with Human Beings at the Federal University of São Carlos, CAAE: 3654240.9.0000.5504 and Brazilian Registry of Clinical Trials (REBEC) RBR—3t85fd, registered on the 25th of September, 2020.

## Background

According to the World Health Organization (WHO) [1], the world is experiencing a rapid and intense change in population patterns and the number of older people is increasing [2]. Given this scenario, there is a need to promote adequate care for this population, through strategies to preserve both functional and cognitive capacity with main focus on quality of life years [3, 4].

One of the main issues related to old age is the occurrence of falls, which may be linked to multiple intrinsic or extrinsic factors [5, 6]. According to the WHO [7], a fall is an unintentional dislocation that results in the body's transition to a position below the initial level, with an inability to correct it in a timely manner. Falls can cause fractures, difficulty in performing activities of daily living (ADL), health declines, injuries, hospitalizations, institutionalization, death, and fear of falling again [7, 8]. In relation to the years lived with disability (YLDs), accidents due to falls occupy the 16th position among people aged 50 to 74 years old and the 8th position among people aged 75 years old and over [9]. This highlights the great need for monitoring faller older people, preventing falls and reducing consequent damages to health. Faller older people demand high targeted care from the health system, mainly due to their need for care at high levels of complexity and specialized services, such as surgical procedures and hospitalizations. This generates increased public health expenses [1, 10, 11]

Cognitive functioning is another important dimension within older people. Maintaining a good memory is vital for aging, due to its association with autonomy and independence. Mood disorders, anxiety and social isolation may be present in the older people's life, compromising health and favoring cognitive decline. Aging can bring functional and cognitive changes. Other common chronic conditions such as depression and cognitive impairment can generate countless losses and possibility of accidents such as falling [12].

Cognitive impairment is an important risk factor for falls. Older people with cognitive impairment have a higher prevalence of falls when compared to cognitively preserved older persons [13]. The higher the cognitive changes and the falls rate, the worse the performance on gait, balance and functionality, which increases risk of falls in faller older people with cognitive impairment [14–16].

There are several mechanisms available in health and social systems that aim at reducing the incidence of falls and their risk factors [17, 18]. Among falls prevention programs, cognitive stimulation can be used, with varied cognitive domains among methods. In a systematic review, the relationship between attention, learning and executive function with gait control and occurrence of falls was evidenced [16, 19]. Thus, programs that stimulate specific cognitive domains can bring more benefits on falls data and risk factors for falls in faller older people with cognitive impairment.

The main evidence for falls prevention is related to physical exercises that challenge balance. However, other falls prevention programs still need to be better evidenced. The number of falls programs aimed at cognitive stimulation is still low, even after the relationship between different domains of cognition, gait and falls have been proved [18, 20]. Considering the importance of preventive programs in older adults’ health, this study aims to share an intervention protocol of a falls prevention program for community-dwelling faller older adults with cognitive impairment.

## Methods/design

This is a 16-week, longitudinal, quantitative, experimental, and single-center study. The assessors will be blinded for randomization, with evaluation before the beginning and after the end of the proposed activities. The cognitive stimulation will be conducted by a gerontology undergraduate student with training and supervision by a gerontologist specialized in the area.

### Participants

Recruitment will be conducted through contact with health services, network professionals and the Research Group's database, with folders, flyers, cards, pamphlets and posters. Social networks, television channels, newspapers and radio will be also used for disclosure.

The sample will be composed of community-dwelling Brazilian older people aged 60 years old and over. Inclusion criteria will be presenting a history of two or more falls in the last twelve months, being available for interventions and being able to walk alone, using or not walking devices. In addition, they should score below the cutoff point on the Mini Mental State Examination according to years of education [21]. Participants will be discontinued from the study if they start another treatment for the management of falls and/or present an absence greater than 25% from activities.

Individuals will be excluded from the study if: the researcher is unable to make prior contact by telephone after three attempts at alternate times and days of the week; the volunteer is not able to answer the evaluation questionnaires; cancer diagnosis; active inflammatory arthritis; having neurological diseases such as stroke, advanced Parkinson's disease (stage 5 of the Hoehn and Yahr scale modified and not being in regular use of antiparkinsonian drugs) or other neurological disorders; uncorrected visual or hearing impairments; and vestibular system disorders.

### Sample calculation

The sample calculation was performed using the statistical program G*Power 3.1. Assuming: 1) the type of study design (two-way ANOVA); 2) the type I error in 5% (α = 0.05); 3) the statistical power at 80% (1-β = 0.80); 4) assuming an effect size of moderate magnitude (0.20); and 5) the number of groups and measures, a minimum of 42 people must constitute the total sample. Considering the chance of loss of 20% of the participants, it is estimated a sample of 60 people in total.

### Procedures

After inclusion of the sample, the first evaluation will take place. After this evaluation, the volunteer will be randomized (block randomization process, 1:1 allocation ratio, by an independent researcher) and sent either to the control group (CG) or experimental group (EG). The researcher responsible for the training will contact him/her and start the period of 16 weeks of intervention. After that, the post-test evaluation will take place. After the reassessment, a feedback will be scheduled, with the goal of informing about participant’s performance in the pre and post test.

### Intervention

**Fig. 1:**
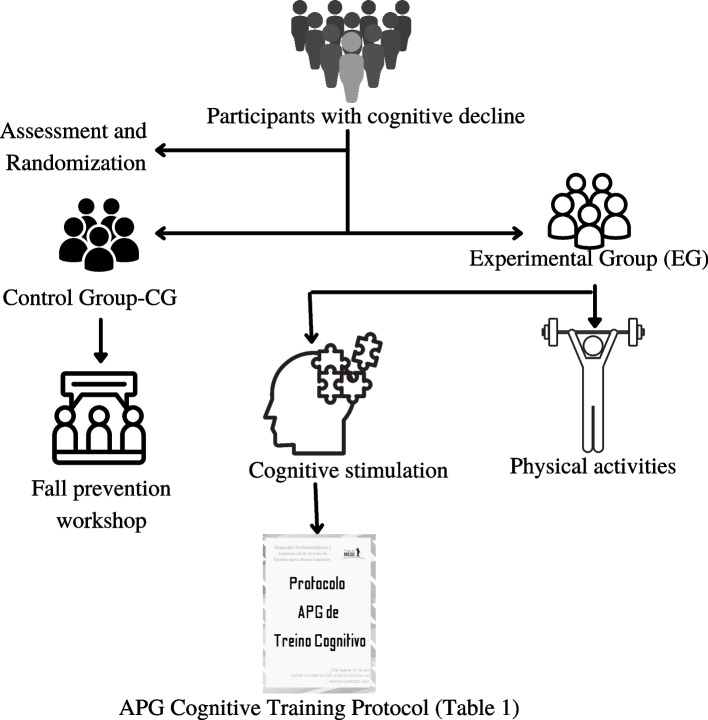
Protocol description

The intervention will initially occur with the randomization of participants into two groups, namely: EG: falls prevention workshop with physical activities and cognitive stimulation. CG: falls prevention workshop (Fig. 1).

Cognitive training assumes a progression in the level of cognitive activities. Over the weeks, they permeate various stimulated cognitive domains, intensifying levels of complexity. Activities that are offered more than once, with complementary guidelines, are considered necessary at both levels offered, as they demand more and more from the participant and are in line with the progressive intention of training.

Contact with the volunteer will take place at a period agreed between the volunteer and the trainer. The first week will also include the presentation of the Protocol and the methodology to be applied, with spaces to solve questions and other issues that arise during implementation. A kit of basic materials (e.g. pencil, eraser and glue) will be provided, as well as the exercise book and auxiliary materials for the activities (e.g. Tangram, puzzles, colored sticks, etc.).

Each intervention consists of a synchronous call to carry out an activity and the details of the activities that should be done for the next call. Each activity has illustrated examples that facilitate understanding. After completion, the volunteers must be sent as photographs in the communication channel with the responsible trainer. When the intervention ends, the participant will be reassessed.

The activities were planned and organized to take place for 16 weeks, and will be based in the APG Cognitive Training Protocol. This protocol consists of a material that contains all the activities. Table 1 shows the detailed information of each week of intervention.

**Table 1:** Activities to be performed weekly according to the cognitive stimulation booklet

Week	Activity	Cognitive domain
1	1- Colored Balloons 2- What's the word? 3- Draw the story	Attention, Memory and Learning, Executive Functioning, Social Cognition, Language
2	1- What is alike? 2- Complete Drawing 3- In which song is that?	Attention, Memory and Learning, Executive Functioning, Perceptual motor, Language
3	1- Count and paint 2- Whose footprint is that? 3- Sing the sayings	Attention, Memory and Learning, Executive Functioning, Social Cognition, Language
4	1- Find the objects 2- Do the drawing 3- Crossword 4- Dual task	Attention, Memory and Learning, Executive Functioning, Language
5	1- Copying the sticks 2- Naming 3- Left or right?	Attention, Memory and Learning, Executive Functioning, Language
6	1- Paste the letter 2- Write the objects 3- Math and results	Attention, Memory and Learning, Executive Functioning, Language
7	1- Find the pairs 2- Maze 3- Paint the pairs 4- Grocery Store	Attention, Memory and Learning, Executive Functioning, Language
8	1- Crossword 2- Painting 3- Finding the words	Attention, Memory and Learning, Executive Functioning
9	1- Finding the objects 2- What’s the contrary? 3- Circle the rhymes 4- Find the pairs	Attention, Memory and Learning, Executive Functioning, Social Cognition, Language
10	1- Copy the images 2- What’s the time? 3- Circle what’s repeated 4- Complete the song	Attention, Memory and Learning, Executive Functioning, Language
11	1- Find the mistakes 2- Route 3- Find the pairs 4- Dual task	Attention, Memory and Learning, Executive Functioning, Language
12	1- Creating words 2- Crossword 3- Maze 4- Connecting the dots	Attention, Memory and Learning, Executive Functioning
13	1- Tangram 2- Finding the mistake 3- What’s next? 4- Filling with the time	Attention, Memory and Learning, Executive Functioning, Perceptual motor
14	1- What’s the intruder? 2- Painting 3- Dual task 4- Folding	Attention, Memory and Learning, Executive Functioning, Social Cognition, Language
15	1- Circle the numbers 2- What’s the relationship? 3- Finding the objects 4- Puzzle	Attention, Memory and Learning, Executive Functioning, Perceptual motor
16	1- Count the images 2- Crosswords 3- Daily activities 4- The colors puzzle	Attention, Memory and Learning, Executive Functioning

#### Weekly description of activities

- Week 1: “Colored Balloons”, consisting of writing objects with the same colors as the illustrated balloons; “What’s the Word?”, with shuffled letters and must be ordered to form a word; “Draw the Story”, encouraging the dual task of telling a story and drawing it simultaneously.
- Week 2: “What is alike?”, in which a symbol is presented and all those that are the same should be circled; “Complete the Drawing” in which drawings half-hidden must be completed; “In which song is that?”, with random words that must be found in some song, listing the name of the song.
- Week 3: “Count e Paint”, to solve addition and subtraction gaps and coloring with the appropriate color; “Whose footprint is that?”, with the objective of identifying which animal owns each footprint and answering 2 questions related to these animals; “Complete the sayings”, consisting of 15 proverbs partially written, to be completed.
- Week 4: “Find the objects”, in an image full of objects, cleaning and hygiene items should be circulated and counted; “Do the Drawing” with gaps for the participant to draw what is requested; “Crossword”, the task is to find words vertically and horizontally, in addition to counting them and describing the relationship between them; “Double Task”, encouraging the performance of a 60-s walk, evoking fruit in the first attempt and fruit starting with the letter “A” in the second.
- Week 5: “Copying the Sticks”: 4 images with colored lines that must be memorized and replicated with colored sticks; “Naming”, to name the images; “Left or right?”: 6 figures of human hands are exposed and must be judged as left or right, also participants are required to write a sentence with the non-dominant hand.
- Week 6: “Paste the letter” with objects placed next to gaps, and the participant must paste the initial letter of each one of them; “Write the Objects”, in which the figures present in an image with several superimposed figures that must be named; “Math and Results”, consisting of two columns, one with subtraction accounts and the other with results.
- Week 7: “Find the Pairs”, with identical objects that must be circled in the same colors, in addition to answering 3 general questions about the objects; “Maze”, to find the way out of the maze; “Paint the Pairs”, in which the pairs of slippers must be identified and customized with the same colors; “Grocery store”, with a list consisting of 15 usual market items and questions to evoke them, classifying them into categories such as hygiene, food and beverages.
- Week 8: “Crossword”, with words in horizontal and vertical, which must be transcribed; “Painting” in which a black and white drawing must be colored, with the association of songs being requested while the volunteer paints; “Finding the words” in which the clues are given, the words must be thought out and written.
- Week 9: “Finding the Objects”, in which 11 objects dispersed in an image must be found; “What’s the Contrary?”, with a list of 20 words whose opposites must be thought out; “Circle the Rhymes”, in which the rhymes in the text must be circled in the same color with their pairs, then answer questions about the text and create two other rhymes; “Find the Pair” with a table of letters whose pairs must be found and circled, then think of 2 words that only use the unpaired letters and 2 words that do not use any unpaired letters.
- Week 10: “Copy the Image”, in which a figure must be copied to the side; “What’s the time?”, with 9 clocks, must note the time set by each one; “Circle what’s repeated”, finding the pairs of each word, also answering if any does not have a pair; “Complete the song”, in which a popular song will be displayed with gaps to be completed.
- Week 11: “Find the mistakes”, with two similar images whose differences must be found; “Route” in which a course must be made on a squared “map” with barriers, after making the route, it must be narrated; “Find the Pairs”, in which scattered letters must be circled in the same colors as their pairs and then form words that do not use the circled letters; “Dual Task”, applied again, during the same period and with evocation of animals and then animals with the letter “C”.
- Week 12: “Creating Words”, in which 8 example words are given and other words that do not have any letter already used in the example word should be thought; “Crossword”, applied again with words in horizontal and vertical, and questions related to the words found; “Maze”, in which the way out of the labyrinth must be found and colored; “Connecting the dots”, consisting of 37 dots that must be connected to form a pattern.
- Week 13: “Tangram” in which the 7 tangram pieces were provided and volunteers must replicate the images; “Finding the mistakes”, in which the differences between the two images must be circled; “What's Next?”, the symbol of the logical sequence must be identified and drawn; “Filling with the time”, in which the hands of the clock must be placed according to the indicated hour;
- Week 14: “What is the intruder?”, in which the “intruder” word must be selected and justified; “Painting”, in which the drawing with gaps must be colored, requesting the evocation of the alphabet during the painting; “Dual Task”, reapplied during the same period and with the evocation of women’s names and then women’s names with the letter “M”; “Folding”, featuring a step-by-step process for making a fold.
- Week 15: “Circle the Numbers”, in which, inside a table, the numbers must be circled according to the coordinates; “What is the relationship?” in which the relationship between each trio of words must be explained; “Find the Objects”, with 8 objects that must be found in the presented image; “Puzzle”, with the assembly of a puzzle and questions about it.
- Week 16: “Count the image”, in which the indicated symbols must be searched for in an image and counted; “Crossword”, applied again with words in horizontal and vertical, with related questions; “Daily Activities”, in which the description of the figures with actions must be performed; “Color Puzzle”, consisting of 9 pieces, which must be positioned properly to meet the requested requirements.

With the completion of this study, it is expected to demonstrate the importance of public health actions that favor, monitor and encourage the mental and physical health of older people, which, according to the World Report on Aging and Health [1], should be carried out in all scenarios, especially in the context of the COVID-19 pandemic. The research has great relevance also because the literature has few studies that detail their protocols and serve as a basis for them to be replicated by professionals in the context of cognitive maintenance for the elderly.

### Outcomes

The primary outcomes are the risk and incidence of falls, as well as cognitive performance. Also, the secondary outcomes are balance, muscle strength, mobility, gait speed, anxiety, instrumental activities of daily living, depressive symptoms, and anxiety.

### Assessment

The included participants will be guided and evaluated through scheduled interviews, in places that ensure the privacy and confidentiality of information. The protocol consists of the following screening instruments:

- Participant Characterization Instrument—composed of questions related to: Identification (name, gender, age); Sociodemographic information (ethnicity, education, marital status); Health conditions (use of medications, associated diseases, self-perception of health, vision, hearing, pain); Basic assessment and self-report of falls, including incidence/history, fear of falling, description of the environment and previous fractures.
- Timed “Up and Go”—TUG: aims to assess balance and mobility. It consists of timing the time taken to get up from a chair, walk 3 m (marked on the ground), rotate, return the same 3 m and again sit leaning against the backrest. The instrument score adopted as a risk of falls for elderly Brazilians is 12.47 s. The modified version of the instrument will also be used, associated with the cognitive task, in which the elderly person must speak, without stopping, names of animals during the course of the simple TUG [22, 23].
- Short Physical Performance Battery—SPPB: aims to assess balance, muscle strength and gait speed. The battery is divided into: balance test, which consists of remaining standing for 10 s in three different positions/difficulties; gait speed test, timing and recording the seconds spent to walk 2 times 3 or 4 m, considering the shortest time between attempts; five times standing up from a chair test, performed after a pretest of standing up. With partial scores at each stage and the participant's ability/speed to perform the activities. The overall cutoff score is the sum of the partial scores of the three dimensions, ranging from 0–3 (poor performance/disability), 4–6 (poor performance), 7–9 (moderate performance) and 10–12 (good performance) [24].
- Geriatric Depression Scale – GDS (15-item version): with the objective of verifying depressive symptoms. The 15 questions have the answer option “yes” or “no”, each question has a score equal to 1. The final score is obtained by the sum of all questions. 0 to 5 points indicate normality, 6 to 10 points for mild depressive symptoms and 11 to 15 points for severe depressive symptoms [23, 25].
- Falls Effectiveness Scale – International – (FES-I): has the purpose of evaluating the fear of falling when performing certain external activities and social participation. The scores for each question range from 1 (not at all concerned), 2 (somewhat concerned), 3 (very concerned), and 4 (extremely concerned). The final score is the sum of the scores, ranging from 16 (no concern) to 64 (extreme concern), with 23 points being the cutoff for sporadic falls and 31 points for recurrent falls [26].
- Assessment of Lawton's activities of daily living: aims at evaluating performance in Instrumental Activities of Daily Living (IADL). The instrument has questions related to telephone use, shopping, travel, preparing meals, housework, using medication and handling money. The total score ranges from 0 to 21 points, and for each criterion, total need for assistance scores 3, partial need scores 2 and no need scores 1. The final score equivalent to 7 points is indicative of total dependence [23, 27].
- Addenbrooke Cognitive Examination—Revised Version (ACE-R): aims to evaluate cognitive global functioning. The instrument consists of the Mini Mental State Examination (MMSE) and an assessment of five cognitive domains, with partial grades, including: attention and orientation (0 to 18 points), memory (0 to 26 points), fluency (0 to 14 points), language (0 to 26) and visual-spatial skills (0 to 16 points). The final score is composed of the MMSE score (0 to 30 points) and the evaluation of the domains, obtained by partial sums, ranging from 0 to 100, the closer to the maximum score, the better the cognitive status [28].
- Digit Span: aims to examine attention, immediate memory and working memory, using the digit repetition feature in forward and reverse order. The range of digits in the forward order checks attention deficit and/or immediate memory (score less than 6), the reverse order checks attention deficit and/or working memory (score less than 4). The digits are divided into seven series, consisting of two to nine digits each.
- Geriatric Anxiety Inventory (GAI): assesses anxiety. Composed by 20 items focused on aspects of anxiety, the answer varies according to whether the individual agrees or disagrees with the statements presented. The score of each answer varies from 0 to 1, in positive answers, 1 point is scored. The final score is obtained by summing the scores, with 10/11 points being the cutoff indicative of the presence of generalized anxiety [29].

All adverse effects of the study will be monitored through contacts with the participants at all times of intervention. Furthermore, all volunteers will be promptly assisted by researchers in case of any intercurrence that may influence their well-being as agreed in the Research Ethics Committee.

### Data treatments and statistical analysis

The collected data will be initially entered to an Excel spreadsheet. Typing will be performed by two independent researchers. Then, they will be exported to the Statistica software program, version 7.0.

The Shapiro wilk test will be performed to verify the normality of the data and then the intergroup statistical analysis (before and after). Descriptive analysis will be conducted to characterize the participants’ profile. For intergroup analysis, the Mann Whitney test will be used for non-parametric data, and the Student’s T test will be used for parametric variables. The significance level adopted for the statistical tests will be 5% (p ≤ 0.05).

The results will be published for the public and scientific community as articles. Only the researchers who took part in the research will have access to the information for future publications.

### Ethical aspects

Participants will be informed about the study procedures and they must complete an electronic form, the Informed Consent Form, consenting their participation. The research was approved by the Research Ethics Committee with Human Beings at the Federal University of São Carlos, CAAE: 3,654,240.9.0000.5504 (approved 22 September 2020) and Brazilian Registry of Clinical Trials (REBEC) RBR—3t85fd (registered 25 September 2020 https://ensaiosclinicos.gov.br/rg/RBR-3t85fd). The information of the elderly will be kept confidential, respecting the ethical guidelines. Identification numbers will be assigned to ensure the confidentiality of participants’ names. The trial will not be stopped in case of futility.

## Discussion

Multimodal cognitive interventions consist of a relevant alternative in the context of health prevention and promotion due to its methodological flexibility, which is able to reach a great number of individuals [30]. Moreover, when it comes to experimental studies, follow-up assessments considerably increase the methodological robustness and provide evidence on long-term effects of interventions [31, 32]. In this sense, this protocol describes a follow-up, experimental study in which a multimodal cognitive intervention is detailed. An interesting aspect of this study is the fact that its participants will be able to fill their free time (e.g. periods of inactivity) with cognitive interventions. In addition to being significant for cognitive performance, the literature suggests such interventions (i.e. multimodal interventions) create bond and motivation for participants' adherence and engagement, which contributes to the effective conclusion [33, 34].

This study will contribute to the scientific literature providing evidence on the effects of the practice of a multimodal cognitive training intervention within a fall prevention program for elderly community fallers with cognitive decline. Its execution will provide information on whether participants who underwent multimodal cognitive stimulation will experience improvements in cognitive functions, balance, mobility, and risk of falls when compared to a control group in the community. It will also seek to assess the consequences of cognitive training on gait speed, presence of depressive symptoms, and anxiety. Robust evidence in the literature suggests the benefits of cognitive stimulation intervention on clinical variables such as cognitive performance [35], mobility [36], gait speed [37], and depressive symptoms [38]. However little is known about the potential effects of cognitive intervention with a multimodal approach in a fall prevention program [39]. Also, to the best of our knowledge, this is the first study which will analyze the proposed intervention for cognitively impaired older adults in a fall prevention program, with a follow-up assessment.

Another relevant aspect of this study is that tracking and monitoring risk factors for falls during the experiment will contribute to a significant and robust mapping and understanding of the relationship between the causes and consequences of falls. It will also evaluate the efficiency of the cognitive intervention guided by the APG protocol and its reproducibility.

The target population of the study represents the age group with the highest growth in the current scenario, which highlights the importance of interventions that focus on maintenance and improvement of mental health [40]. In spite of that, one of the biggest challenges in old age is the maintenance of cognitive capacity, since individuals with cognitive decline tend to have negative consequences in their performance of activities of daily living (ADL) and independence [41]. In this sense, multimodal cognitive interventions also seem to positively impact older adults' ADLs and independence [37, 42].

Interventions through supervised activities also generate benefits by providing low application costs and high comprehensiveness of participants, as well as by requiring less monitoring from family members or caregivers [30]. This protocol may support not only researchers, but also health professionals in their clinical practice. On the same hand, they will be able to benefit both their older adult patients and their families/caregivers.

Finally, in a post-pandemic scenario, remote activities play increasing roles in the context of telemedicine. It, in turn, aims to eliminate physical barriers in the provision of care (e.g. distance and transportation to health facilities), and proposes the effectiveness of health promotion, whose focus is on the population [43]. In this sense, remote multimodal cognitive interventions are a promising strategy for improving cognitive functions, as they allow the creation of personalized and optimized training, in a simplified and accessible way, with greater cost benefit when compared to traditional interventions [30].

## Data Availability

Not applicable.

## Abbreviations

ACE-R: Addenbrooke Cognitive Examination—Revised Version
ADL: Activities of Daily Living
CG: Control Group
EG: Experimental Group
FES-I: Falls Effectiveness Scale – International
GAI: Geriatric Anxiety Inventory
GDS: Geriatric Depression Scale
IADL: Instrumental Activities of Daily Life
MMSE: Mini Mental State Examination
REBEC: Brazilian Registry of Clinical Trials
SPPB: Short Physical Performance Battery
TIG: Timed “Up and Go”
YLDs: Years Lived with Disability
WHO: World Health Organization
